# Predicting isocitrate dehydrogenase status in glioma using hierarchical attention-based deep 3D multiple instance learning

**DOI:** 10.3389/fonc.2025.1665690

**Published:** 2025-12-18

**Authors:** Qinqin Xie, Yongheng Sun, Yuxia Liang, Yu Shang, Haifeng Wang, Fan Wang, Rong Wei, Bin Chen, Ming Zhang, Chen Niu

**Affiliations:** 1 PET-CT Center, The First Affiliated Hospital of Xi’an Jiaotong University, Xi’an, China; 2 Department of Network Information, The First Affiliated Hospital of Xi’an Jiaotong University, Xi’an, China; 3 School of Mathematics and Statistics, Xi’an Jiaotong University, Xi’an, China; 4 Data Center, Hangzhou First People’s Hospital, Hangzhou, China

**Keywords:** dynamic gated attention, glioma, IDH, location encoding, Multiple Instance Learning

## Abstract

**Background:**

According to the 2021 WHO classification of tumors of the central nervous system, isocitrate dehydrogenase (IDH) status serve an independent prognostic biomarker and is closely associated with tumor diagnosis and treatment response. At present, the determination of IDH status still relies on invasive surgical procedures.

**Method:**

A total of 345 patients with pathologically confirmed gliomas diagnosed at the First Affiliated Hospital of Xi’an Jiaotong University between October 2019 and October 2024 were retrospectively included, comprising 148 (42.9%) IDH-wild and 197 (57.1%) IDH-mutant. An additional 495 glioma patients were obtained from the public TCIA dataset. Patients were randomly split into training, validation, and test cohorts 6:2:2. A Hierarchical Attention-Based Multiple Instance Learning (HAB-MIL) framework was developed, integrating auxiliary positional encoding into feature maps to capture spatially specific information and generate refined 3D lesion representations. Model performance was evaluated using five-fold cross-validation, with receiver operating characteristic (ROC) curves, area under the curve (AUC), sensitivity, and specificity as assessment metrics.

**Result:**

HAB-MIL achieved competitive performance, with AUCs of 0.917 and 0.892 on the glioma datasets from TCIA and the First Affiliated Hospital of Xi’an Jiaotong University. Additionally, our work achieves results that are comparable to the state-of-the-art methods in TCIA dataset and demonstrates that multiple instance learning has great potential for IDH prediction.

**Conclusion:**

The proposed HAB-MIL achieved IDH classification based on conventional preoperative MRI images, eliminating the need for pixel-level annotations and significantly reducing the annotation burden for doctors.

## 1 Introduction

Gliomas, the most prevalent malignant tumor in the central nervous system, account for approximately 80% of all intracranial tumors (1, 2). According to the WHO 2021 classification of central nervous system tumors, isocitrate dehydrogenase (IDH) status is considered as a critical biomarker for the diagnosis and treatment of glioma and plays a vital role in prognosis and treatment strategies (3, 4). Previous studies have shown that patients with IDH-wild generally have a poorer prognosis and exhibit less sensitivity to the therapeutic targeting of lDH mutations with vorasidenib compared with those with IDH-mutant (5–7). At present, determination of IDH status primarily depends on surgical sampling followed by genetic sequencing. However, these approaches have several limitations: lesions that are deeply situated or located near eloquent brain regions may be difficult or impossible to sample (8, 9). Consequently, the accurate and noninvasive prediction of IDH status has become an urgent clinical need.

Recent advances in artificial intelligence, particularly in deep learning, have enabled noninvasive assessment of IDH status through MRI (10–12). Deep learning models can automatically learn complex patterns directly from raw images, thereby minimizing the need for manual feature extraction and extensive domain expertise. In contrast to traditional tissue biopsies, it provides a safe, efficient, and repeatable way to preoperative evaluation and long-term follow-up (13, 14). However, it largely depends on access to high-quality annotated datasets (15). Data labeling is labor-intensive and requires the expertise of highly trained specialists.

To overcome the challenges, weakly supervised learning approaches—such as Multiple Instance Learning (MIL)—have gained considerable attention in medical image analysis (16–18). In the MIL framework, an image is regarded as a “bag” consisting of multiple “instances,” such as image patches or slices. For binary classification tasks, a bag is labeled as positive if at least one instance is positive. By relying on bag-level rather than instance-level labels, MIL is well suited for medical imaging lacking of fine-grained labeling (19).

Although MIL approaches, such as Attention-based Multiple Instance Learning (AB-MIL) and Clustering-constrained Attention Multiple Instance Learning (CLAM), have achieved remarkable progress in instance-level feature aggregation, they often overlook the spatial positional relationships among instances within the original image. In AB-MIL, the attention weights are derived from a fixed parameter matrix shared across all samples. This global static attention mechanism struggles to adapt to the diverse morphological variations of lesions and tends to overemphasize irrelevant background or noisy regions (20, 21). Similarly, CLAM, despite introducing a clustering constraint to improve instance-level discriminability, still relies on a single static attention mechanism and therefore lacks the capability to dynamically adjust attention distributions in response to varying feature patterns across different samples (22, 23).

To address these limitations, we propose a MIL network named Hierarchical Attention-Based Multiple Instance Learning (HAB-MIL). The model includes a Dynamic Gated Attention (DGA) module that integrates a learnable singular value decomposition (LSVD) framework. This framework adaptively decomposes and reweights the instance feature matrix, allowing dynamic modulation of the key components within the attention map. It enables the network to capture latent inter-instance dependencies and adjust the attention distribution in a data-adaptive manner.

## 2 Method

### 2.1 Patient

**Figure 1:**
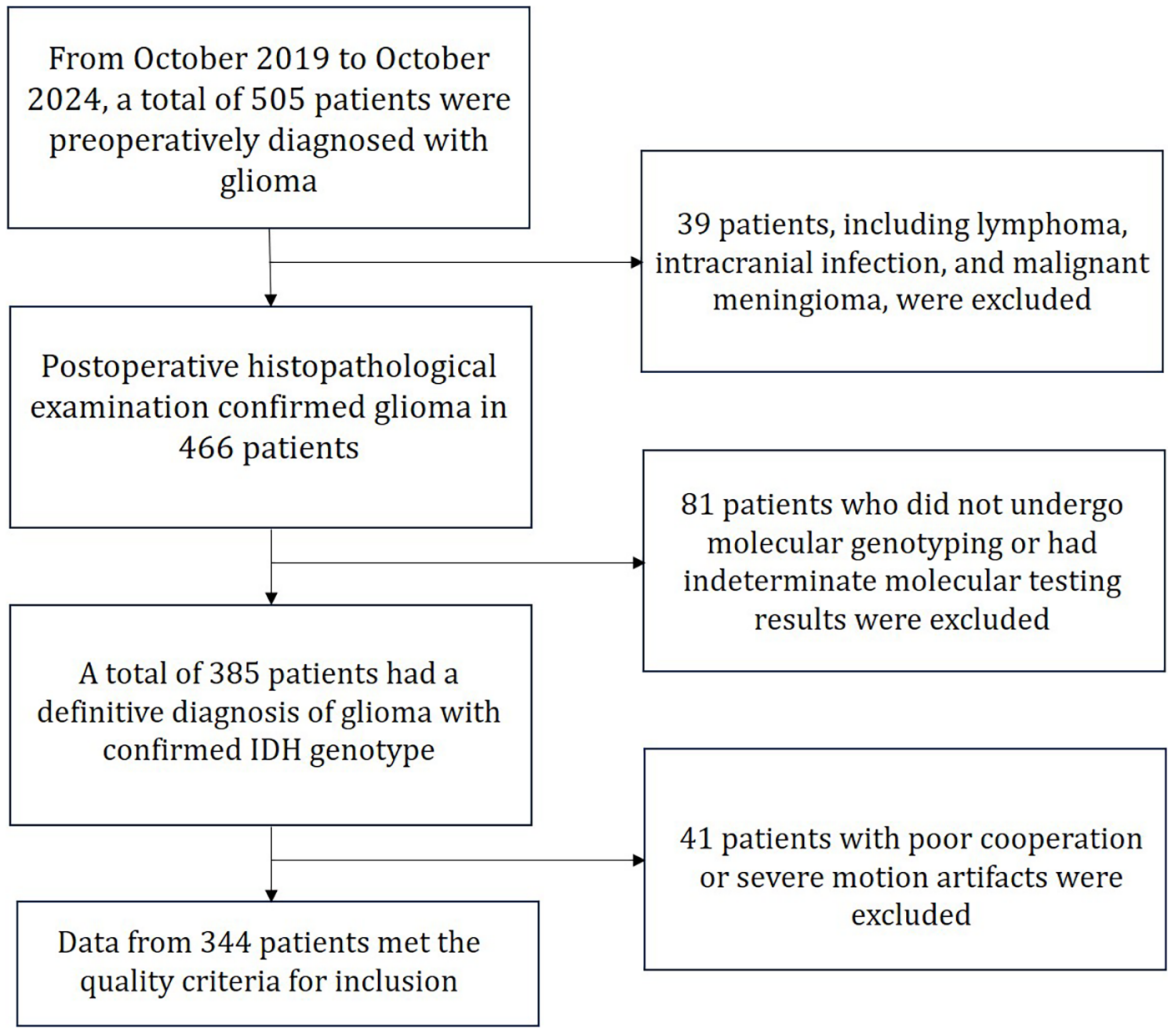
Flowchart of patient inclusion and exclusion.

This study included patients who were preoperatively diagnosed with glioma and underwent surgical treatment at the Department of Neurosurgery, the First Affiliated Hospital of Xi’an Jiaotong University, between October 2019 and October 2024. A total of 506 patients with histopathologically confirmed glioma and available genetic testing were initially screened according to the 2021 WHO classification of tumors of the central nervous system, 5th edition. After applying the inclusion and exclusion criteria, 345 patients who met all requirements were ultimately enrolled in the study cohort, as shown in **Figure 1**. The IDH status of all data was determined by DNA sequencing.

Inclusion Criteria:

1. Patients aged 18–80 years with a single intracranial lesion confirmed as glioma by postoperative pathology, with complete molecular genetic testing results.
2. Mini-Mental State Examination (MMSE) score between 28 and 30, indicating no significant cognitive impairment and sufficient ability to cooperate during MRI scanning.
3. No MRI contraindications, such as metallic implants, cardiac pacemakers, or severe claustrophobia.
4. No prior history of craniotomy or radiotherapy.

Exclusion Criteria:

1. Poor imaging quality or severe artifacts resulting in data unsuitable for analysis.
2. Presence of infectious, structural, or metabolic brain diseases that could interfere with image interpretation.
3. Incomplete or indeterminate pathological and molecular genetic testing results.

All patients underwent preoperative cranial MRI examinations and provided written informed consent prior to imaging. The study was approved by the Ethics Committee of the First Affiliated Hospital of Xi’an Jiaotong University and was registered at ClinicalTrials.gov (registration number: NCT05019196).

### 2.2 Method

**Figure 2:**
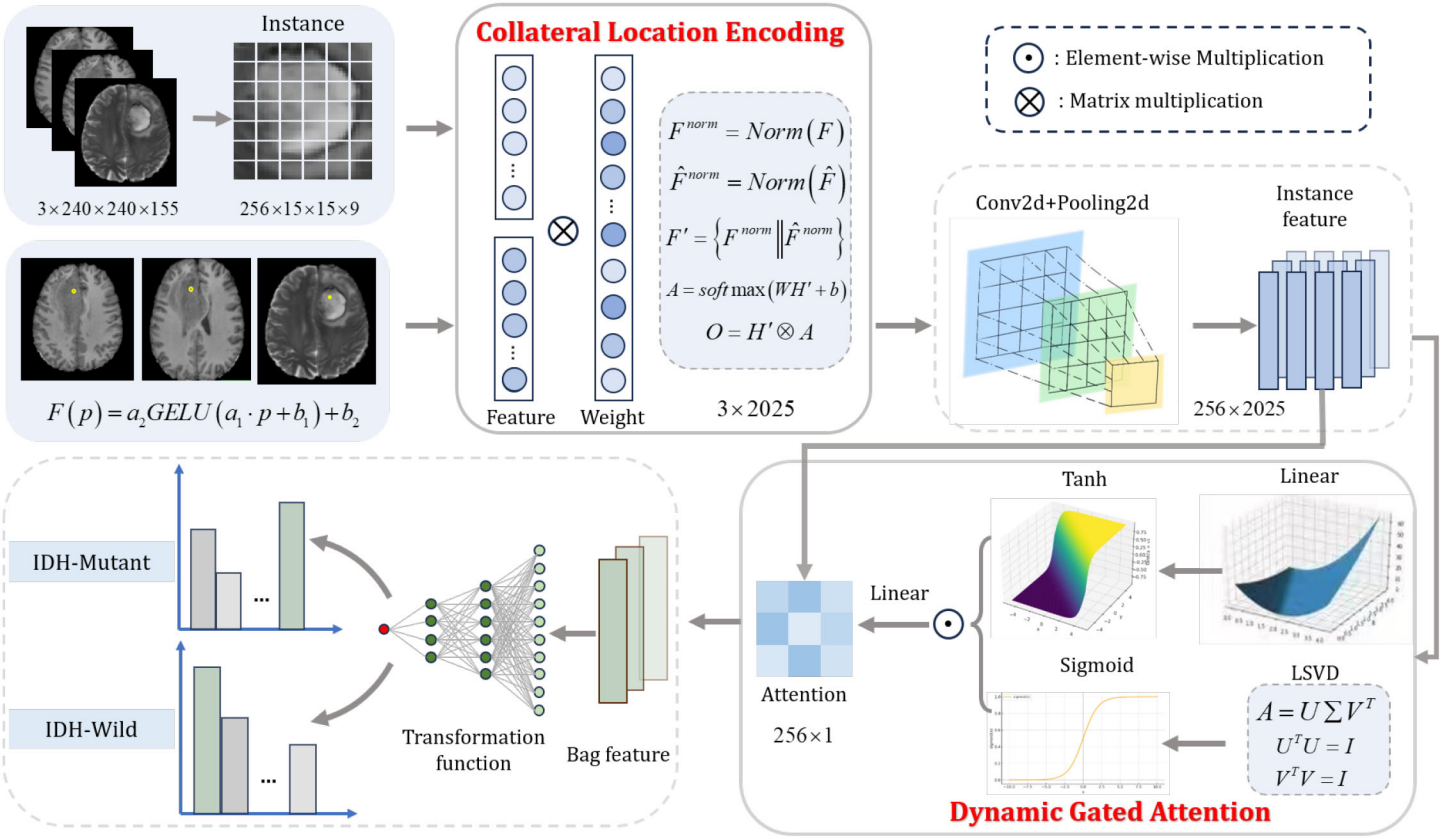
Overview of the hierarchical attention-based multiple instance learning for predicting isocitrate dehydrogenase status in glioma.

Given a complete histopathological image slice X, our goal is to predict the image label Y by analyzing the features extracted from discrimination patches $x_{1},x_{2},\ldots ,x_{n}$. To achieve this, we designed a two-stage framework for the classification of IDH status, as illustrated in **Figure 2**. In the first stage, we encode the spatial features of the tumor. In the second stage, we introduce a novel HAB-MIL network, which comprises multiple layers of convolutional neural networks and an attention mechanism module. This module aggregates the features of the candidate instance in the Yimage-level prediction by recalibrating the importance coefficients for each instance.

### 2.3 Deep instance generation

In MIL, the data is organized into “bags”, each containing multiple “instances”. The label assigned to a bag depends on the instances it contains. For a binary classification task, the bag is labeled positive if at least one instance within it is positive; otherwise, it is labeled negative. In the weakly supervised histopathology image classification problem, the dataset $D=\left\{X_{1},X_{2},\ldots ,X_{N}\right\}$ consists of NMRI images, where each slide $x_{i}$has label $y_{i}\in \left\{0,1\right\}$, and the objective is to train a model to predict the labels of MRIs. This relationship is formally expressed as (Equation 1):

(1) $$Y=\left\{ \begin{array}{l} 0,if\sum_{i=1}^{M}y_{i}=0\\ 1,else \end{array}\right.$$

where Y is the bag label, $y_{i}$ is the label of instance, and is the number of instances in each bag. In our work, this assumption indicates that MRI is from a glioma patient if it involves at least one lesion. Based on the assumption, the empirical loss is formulated by (Equation 2):

(2) $${\hat{Ê}}_{Q}\left(h_{f}\right)=\frac{1}{m}\sum_{i=1}^{m}l\left(h_{f}\left(X_{i}\right),Y_{i}\right)$$

where $h_{f}$ represents a labeling function induced an MIL scoring function f, and l(·)can be any loss function. The MIL process can be mathematically represented as follows (Equation 3):

(3) $$Y=g\left(h\left(f\left(x_{1}\right),f\left(x_{2}\right),\ldots ,f\left(x_{N}\right)\right)\right)$$

where f is the feature extraction function, his the instance-level aggregation operator, g is predicts the bag-level label. The model processes three MRI modalities: T1w, T2w and Flair, which represented as a 3D tensor $x\in R^{B\times C\times H\times W\times D}$, where Bis the batch size, Cis the number of channels and H×W×Dis the spatial resolution. The final layer of 3D fully CNN outputs a series of 3D feature maps $F_{final}$ with the shape of $H^{*}\times W^{*}\times S^{*}\times D$, where $H^{*}$, $W^{*}$, $S^{*}$ and Drepresent the high, width, spatial, and feature dimension of 3D feature maps, respectively. The feature extraction process consists of multiple 3D convolutional layers, batch normalization, ReLU activation, max pooling, and dropout layers. Finally, feature map is formulated as (Equation 4):

(4) $$F_{final}=Dropout\left(MaxPool\left(\sigma \left(BN\left(W_{0}*x+b_{0}\right)\right)\right)\right)$$

where $W_{0}$ is a 3D convolution kernels, with size 3×3×3, and the output channels are 32. ∗indicates the 3D convolution operation. Batch Normalization (BN) is used to stabilize training. σis the ReLU activation function.

### 2.4 Collateral location encoding

The spatial location of a tumor often provides critical diagnostic cues (24, 25), since we proposed Collateral location encoding (CLE), as illustrated in **Figure 2**. P(x,y,z)represents the voxel coordinates of the image. Since p=(x,y,z)typically takes continuous values, employing a smooth nonlinear transformation like GELU allows for a more natural modeling of the relationships between coordinates, unlike ReLU, which introduces abrupt discontinuities. The location feature map F(p)or each position of pixel p=(x,y,z) is calculated as (Equation 5):

(5) $$\hat{F}\left(p\right)=a_{2}\cdot GELU\left(a_{1}\cdot p+b_{1}\right)+b_{2}$$

where $a_{1},a_{2},b_{1},b_{2}$ are the learnable weights and biases of our neural positional encoding layers. The learned features F and location encoding $\hat{F}$from the previous feature extraction are first normalized to benefit the training and backpropagation process. Then features concatenated to be the input of this phase, which can be denoted as (Equation 6):

(6) $$F^{\prime }=\left\{Norm(F)\|Norm(\hat{F})\right\}$$

where ‖means the concatenation operation, Norm(F), $Norm(\hat{F})$ refer to the normalization of F, $\hat{F}$, $H^{\prime }=\left\{H^{norm}\|{\hat{H}}^{norm}\right\}=\left\{\left[H_{1}^{norm}\|{\hat{H}}_{1}^{norm}\right],\ldots ,\left[H_{k}^{norm}\|{\hat{H}}_{k}^{norm}\right]\right\}$ $H_{k}^{norm},{\hat{H}}_{k}^{norm}\in R^{n}$. Then, using the self-attention mechanism, the importance of the features can be calculated by (Equations 7, 8):

(7) $$A=softmax\left(WH^{\prime }+b\right)$$

(8) $$O=H^{\prime }⊗A$$

where W,b denote the learnable weight and bias parameters. $H^{\prime }$and O represent the input and output of the adaptive feature importance weighting mechanism based on the attention module. Softmax operation is applied to normalize the attention scores and resulting in an attention weight matrix A that maps each weight to the interval [0,1]and guarantees the sum of these mapped attention weights to be 1. ⊗denotes element-wise multiplication. Through this process, the input features can be weighted and assigned different importance, enabling the model to emphasize critical features and thereby enhance overall performance.

### 2.5 Dynamic gated attention

Attention mechanisms are well known for dynamically highlighting important parts of input data by assigning different weights, allowing the model to focus on the most critical information for the task at hand (26–28). To enhance the accuracy of learning attention weights, we propose Dynamic Gated Attention (DGA), which achieves superior performance. As illustrated in **Figure 2**, the DGA module integrates two different processing methods.

The first component is an attention representation that uses Tanh as the activation function. With an output range of [−1,1], Tanh provides both positive and negative activation signals, allowing the model to capture complex relationship features. In addition, a dropout function is incorporated to mitigate overfitting. The attention weights are defined as (Equation 9):

(9) $$a_{Tanh}\left(x\right)=\frac{e^{x}-e^{-x}}{e^{x}+e^{-x}}$$

**Figure 3:**
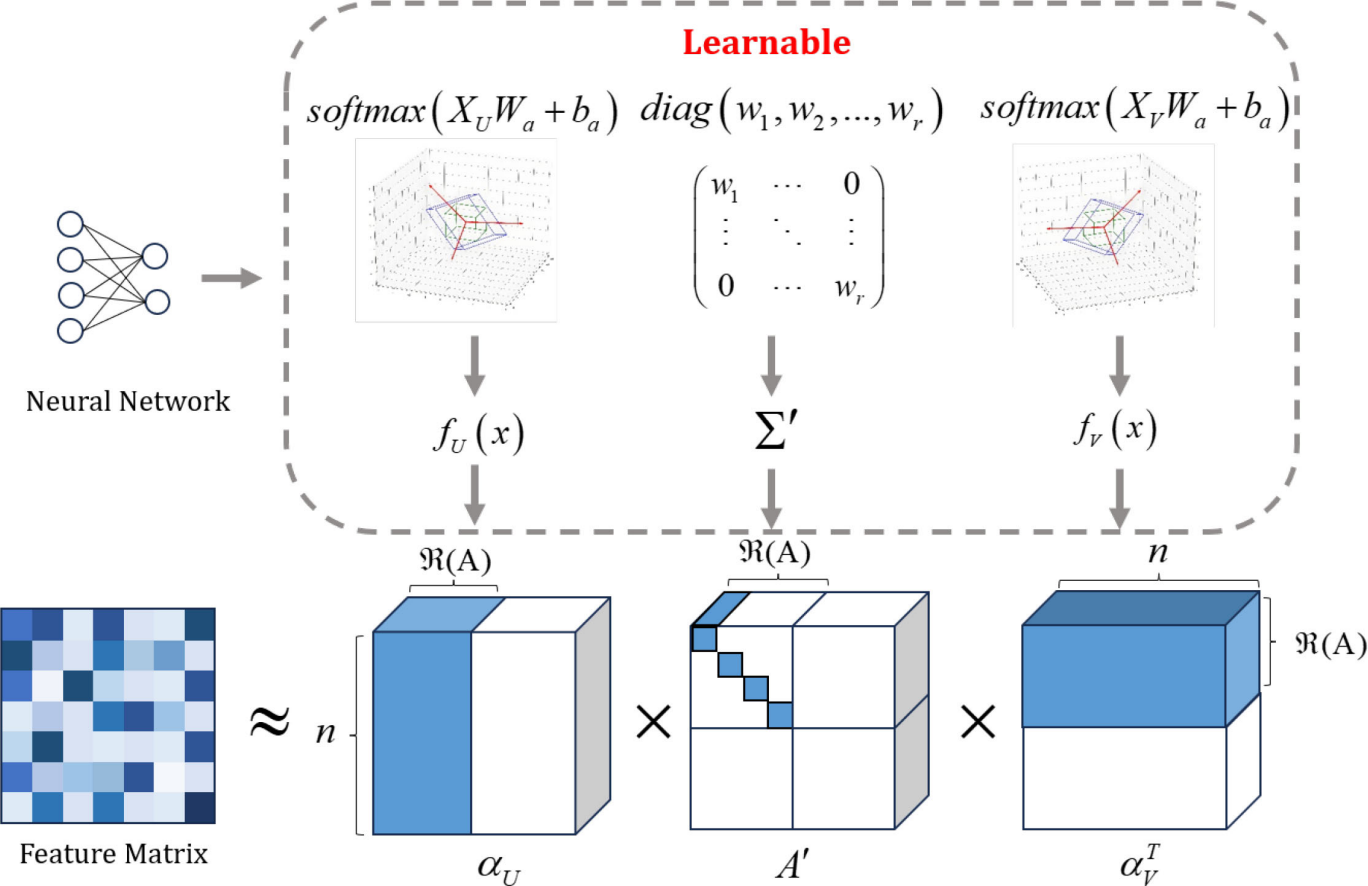
Illustration of the learnable singular value decomposition.

The second component utilizes a different attention representation. As illustrated in **Figure 3**, we propose using Learnable Singular Value Decomposition (LSVD) to reduce the dimensionality of the feature map (29), followed by a Sigmoid activation function, which creates a “switch-like” effect, indicating whether the model should attend to or ignore a particular feature. The algorithm flowchart is shown in **Algorithm 1**.

Algorithm 1 **Learnable Singular Value Decomposition.**

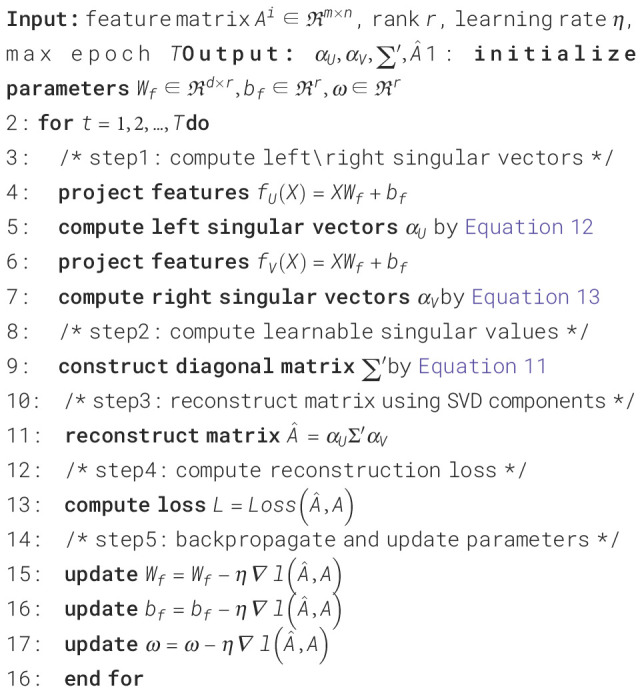

Fact1 The feature map $A^{i}$ can be decomposed as $A^{i}=\alpha_{U}\Sigma^{\prime }\alpha_{V}$, where $\alpha_{U}\in R^{n\times ℜ\left(A^{i}\right)}$ is an orthogonal matrix with the left singular vector of $A^{i}$ as the column vector, where $\alpha_{V}^{T}\in R^{n\times ℜ\left(A\right)}$ is an orthogonal matrix formed by the right singular vectors of $A^{i}$. The matrix $\sum^{\prime }\in R^{ℜ\left(A^{i}\right)\times ℜ\left(A^{i}\right)}$ is a diagonal matrix consisting of the singular values of $A^{i}$.

Proof of the Fact 1: According to the Singular Value Decomposition (SVD), any matrix $M\in R^{m\times n}$ admits a factorization of the form $M=U\sum V^{T}$, where $\sigma_{1}\geq \sigma_{2}\geq \ldots \geq \sigma_{r}>0$ are the singular values and r=ℜ(M) is the rank of M. Hence, for $A^{i}\in R^{m\times n}$, the decomposition is as shown in (Equation 10):

(10) $$\begin{array}{l} A^{i}={\left(\begin{array}{ccc} \mu_{1} & \cdots & \mu_{m} \end{array}\right)}_{m\times m}{\left(\begin{array}{cccc} \sigma_{1} & \cdots & 0 & 0\\ \vdots & \ddots & \vdots & \vdots \\ 0 & \cdots & \sigma_{r} & 0\\ 0 & \cdots & 0 & 0 \end{array}\right)}_{m\times n}{\left(\begin{array}{ccc} v_{1} & \cdots & v_{n} \end{array}\right)}_{n\times n}^{T}\\ \approx {\left(\begin{array}{ccc} \mu_{1} & \cdots & \mu_{r} \end{array}\right)}_{r\times r}{\left(\begin{array}{ccc} \sigma_{1} & \cdots & 0\\ \vdots & \ddots & \vdots \\ 0 & \cdots & \sigma_{r} \end{array}\right)}_{r\times r}{\left(\begin{array}{ccc} v_{1} & \cdots & v_{r} \end{array}\right)}_{r\times r}^{T} \end{array}$$

Here, $\mu_{i},\nu_{i}\in R^{n\times 1}$ are the singular vectors, $r=ℜ\left(A^{i}\right)$is the number of nonzero singular values. For clarity, we denote the left singular matrix $\left(\begin{array}{ccc} \mu_{1} & \ldots & \mu_{r} \end{array}\right)$ as $\alpha_{U}$, the diagonal matrix $diag\left(\sigma_{1},\sigma_{2},\ldots ,\sigma_{r}\right)$ as $\Sigma^{\prime }$, and the right singular matrix ${\left(\begin{array}{ccc} \nu_{1} & \ldots & \nu_{r} \end{array}\right)}^{T}$ as $\alpha_{V}^{T}$, yielding: $A^{i}=\alpha_{U}\Sigma^{\prime }\alpha_{V}^{T}$.

The remaining challenge is to determine $\alpha_{U},\Sigma^{'},\alpha_{V}^{T}$. A natural approach is to leverage neural networks to learn these latent representations. Specifically, assuming $\sum^{\prime }$is a trainable diagonal matrix defined as (Equation 11):

(11) $$\sum^{\prime }=diag\left(w_{1},w_{2},\ldots ,w_{r}\right)$$

where $w_{i}\in R$ are trainable parameters in the neural network, which are initialized prior to training and subsequently optimized via backpropagation.

Then, we utilize two learnable layers of X to fit $\alpha_{U},\alpha_{V}^{T}$ as shown in Equation 12, 13):

(12) $$\alpha_{U}=softmax\left(f_{U}\left(X\right)\right)$$

(13) $$\alpha_{V}^{T}=softmax(f_{V}\left(X\right))$$

Among these, $f_{U}\left(\cdot \right),f_{V}\left(\cdot \right)$ are projection layers to transform the n×d matrix Xinto matrices of shape n×r. In our implementation, we share the same set of two linear layers for $f_{U}\left(\cdot \right),f_{V}\left(\cdot \right)$ by employing a common set of linear transformation parameters $W_{a}\in R^{d\times r},b_{a}\in R^{1\times r}$. The transformation is defined as (Equation 14):

(14) $$f_{U}\left(x\right)=f_{V}\left(x\right)=XW_{a}+b_{a}$$

Formally, we denote $Y=\left\{y_{1},y_{2},\ldots ,y_{n}\right\}$ by a bag of n deep instance, the attention- based MIL pooling is defined by as shown in (Equations 15, 16):

(15) $$a_{n}=softmax\left(Linear\left(a_{LSVD}\cdot a_{Tanh}\right)\right)$$

(16) $$z=\sum_{n=1}^{N}a_{n}y_{n}$$

where $a_{LSVD}$ and $a_{Tanh}$ are attention weights.

## 3 Experiment

### 3.1 Dataset

In this study, two distinct datasets were used for model development and evaluation. Both datasets extract the same features and do not specify whether the IDH-mutant are of the IDH1 or IDH2 type.

In-house data set: The first data set was acquired from the First Affiliated Hospital of Xi’an Jiaotong University and comprised 789 MRI samples of 344 subjects (incorporating FLAIR, T1w, and T2w sequences, all occurrences of “T1w” refer to T1 without contrast injection, and “T1c” refers to T1 with contrast injection.), as summarized in **Table 1**. All patients underwent surgical resection followed by genetic sequencing to confirm IDH status. For patients diagnosed before 2021, we performed a centralized histopathological review. Two board-certified neuropathologists, who specialize in brain tumor pathology, re-examined the archival hematoxylin–eosin and immunohistochemistry slides (including IDH1 R132H, ATRX, p53 and other markers when available) and reviewed the original molecular pathology reports. Based on these materials, tumors were reclassified according to the 2021 World Health Organization Classification of Tumors of the Central Nervous System (5th edition), using all available molecular markers. Importantly, no additional molecular testing (such as TERT, EGFR or chromosomal analyses) was performed because of the retrospective design and limited archival tissue in some case (30, 31).

**Table 1:** Summary of datasets.

Dataset	TCIA		Xi’an	
IDH status	Mutant	Wild	Mutant	Wild
Number	100	395	147	197
Age	38.80 ± 15.11	61.54 ± 15.00	47.58 ± 9.68	52.74 ± 11.97
Gender(M\F)	47\153	234\161	90\57	113\84
Astrocytoma, IDH-mutant	87(17.9%)	\	100(29.0%)	\
Astrocytoma, IDH-wildtype	\	24(4.7%)	\	17(4.9%)
Astrocytoma, IDH-wildtype, NOS	\	\	\	53(15.4%)
Oligodendroglioma	13(2.5%)	\	47(13.6%)	\
Glioblastoma	\	371(74.6%)	\	99(28.7%)
Diffuse midline glioma	\	\	\	28(8.1%)
WHO grade 2	46	10	111	18
WHO grade 3	29	14	36	6
WHO grade 4	25	371	\	173

Public data set: The second data set was obtained from The Cancer Imaging Archive (TCIA), which included 1485 MRI samples from 495 subjects (395 wild-type cases and 100 mutant-type cases). Each subject in the TCIA dataset provided Flair, T1w, and T2w modalities, together with documented IDH status. Both datasets underwent identical pre-processing and post-processing pipelines. The diagnosis of glioma for all patients was performed according to the 2021 WHO classification of tumors of the central nervous system, 5th edition (32). Specifically, MRI volumes were preserved in the DICOM format and standardized to a uniform dimension of 240×240×155. Standard intensity normalization and artifact removal procedures were applied, followed by data augmentation strategies such as color jitter and random affine transformations to mitigate overfitting. This unified preprocessing approach allowed for direct comparisons between the two datasets and a robust evaluation of the proposed HAB-MIL framework.

### 3.2 Implementation details

The framework was implemented using Pytorch2.5.1, and all models were trained with NVIDIA 3090 GPU with CUDA 11.8. We use 3D convolutional neural networks as the deep instance generator. We set the output shape $H^{*}\times W^{*}\times S^{*}\times D$ of ψto be 2×2×2×64 according to cross-validation. The input shapes of the MRI slices are 240×240×155 We set training epoch T to 100 and batch size to 2. Data enhancement strategies included color jitter and random affine transformations. The network was trained using the ADAM optimizer with an initial learning rate 1e-4 and weight decay of 1e-5. In all experiments, 60% of the subjects was used for training, 20% for model selection and hyperparameter tuning, and the remaining 20% for testing. The dataset was augmented with random flipping, random affine transformation, intensity scaling, color jitter as was done to train the segmentation network. No scans from the same subject were included in both the training and the testing samples to ensure data independence and avoid potential information leakage. Five-fold cross-validation is performed within the training set, where the validation portion in each fold is applied for model selection and hyperparameter verification. Each experiment was repeated five times to ensure fair comparisons. Evaluation metrics included accuracy (Acc), area under the curve (AUC), sensitivity (Sens), specificity (Spec) and ROC curves.

## 4 Result

### 4.1 Ablation experiment

A series of ablation studies on the TCIA data set was carried out to evaluate the effectiveness of the two modules (CLE and DGA) in HAB-MIL. Initially, use only the 3D convolution and MIL modules (denoted as Ablation-1) to evaluate the performance of the backbone. In the backbone, features can still be extracted from three concatenated conventional magnetic resonance sequences for the prediction of IDH. Next, add only the CLE module to the backbone (denoted Ablation-2), using positional encoding as a feature, and train the model. Next, add only the DGA module to the backbone (denoted Ablation-3). The results of the ablation experiments are presented in **Table 2**. Compared to backbone results, the incorporation of tumor location encoding improved the accuracy of IDH prediction by 21.8%. This suggests that positional encoding enables our HAB-MIL to learn location-specific features, which aids in predicting consistent differences across all image regions. Then, by adding the DGA module and selecting key instances, the model’s accuracy was further improved by 24%. Finally, combining all three modules results in the best prediction performance. Therefore, the CLE module can be used to explore the spatial relationships of pixels, and the DGA module helps select key instances, improving IDH prediction.

**Table 2:** Classification results in the TCIA dataset of different modules in terms of AUC, ACC, SEN, SPE.

Benchmark	Backbone	CLE	DGA	AUC	ACC	SEN	SPE	Parameter size (M)	Train time (h)
Ablation-1	✓			0.532 ± 0.021	0.514 ± 0.103	0.587 ± 0.015	0.612 ± 0.001	1.165	3.84
Ablation-1	✓	✓		0.750 ± 0.027	0.692 ± 0.001	0.750 ± 0.038	0.699 ± 0.018	1.198	5.83
Ablation-1	✓		✓	0.772 ± 0.007	0.712 ± 0.003	0.781 ± 0.012	0.727 ± 0.003	1.255	5.67
HAB-MIL	✓	✓	✓	**0.917 ± 0.015**	**0.904 ± 0.001**	**0.858 ± 0.008**	**0.921 ± 0.016**	1.313	7.31

Data are given as mean ± SD.

AUC, area under the curve; SEN, sensitivity; ACC, accuracy; SPE, specificity. All values in bold represent the optimal performance.

It applies to all tables unless otherwise noted.

**Table 3** lists classification results of various methods for the prediction of IDH. Among the various methods compared, the SPE can be used as a baseline to measure the validity of segmentation. We first ablate the effects of our CLE in the HAB-MIL for the test. As can be seen, Sinusoidal Positional Encoding (SPE) which simply concatenates the sinusoidal positional encoding of the pixel locations (x, y) values directly into the encoder stage leads to a slight performance improvement of 2% in most metrics compared to those without it (denoted as “w/o SPE”). Bello propose neural positional encoding (NPE) for depth estimation (33). However, our HAB-MIL shows substantial performance improvements in all metrics by incorporating our CLE. Specifically, the AUC increased by 28.8% and the ACC by 27.1% compared to the model w/o SPE. This improvement can be attributed to the learnable auxiliary positional encoding, which allows the model to more flexibly capture and leverage position-specific feature information. From the comparison between these methods, it can be observed that CLE is more helpful for IDH prediction, as it incorporates information that accurately describe location and morphological features of tumor and peritumoral edema.

**Table 3:** Comparison results among CLE module and different location coding functions in terms of AUC, ACC, SEN, SPE.

Method	AUC	ACC	SEN	SPE
w/o SPE	0.629 ± 0.003	0.633 ± 0.005	0.618 ± 0.013	0.607 ± 0.001
SPE	0.631 ± 0.025	0.618 ± 0.018	0.653 ± 0.001	0.593 ± 0.035
NPE	0.863 ± 0.001	0.813 ± 0.012	0.792 ± 0.008	0.901 ± 0.012
CLE	**0.917 ± 0.015**	**0.904 ± 0.001**	**0.858 ± 0.008**	**0.921 ± 0.016**

All values in bold represent the optimal performance.

To evaluate the effectiveness and feasibility of the DGA module within HAB-MIL, we considered three attention weighting methods: max pooling, average pooling, and AB-MIL approaches. These methods were compared against our proposed HAB-MIL model. Specifically, the max-pooling-based and average-pooling-based methods follow the traditional MIL assumption, where the final subject-level prediction is obtained from the most significant instance or the average of all instances within a bag, respectively. In contrast, the attention-pooling-based method leverages an attention mechanism to assign weights to each instance embedding, facilitating learning at the bag level. In contrast, HAB-MIL considers a dynamic attention mechanism to selectively focus on the most relevant instances, effectively minimizing the inclusion of irrelevant information. As shown in **Table 4**, our proposed HAB-MIL achieved the best overall performance, attaining an accuracy of 90.4%. Max-pooling demonstrated the lowest performance among all methods, with an AUC of 69.3%, which is expected as it only considers the single most prominent instance for prediction. In contrast, the mean-based approach, while accounting for all instances, introduces a significant amount of irrelevant information, leading to less satisfactory results. Moreover, although plain attention pooling improved AUC and ACC by 5.3% and 8.9% respectively at the instance level compared to traditional methods, our proposed HAB-MIL achieves superior performance in terms of accuracy, recall, precision and F1, indicating that the effectiveness of the DGA module.

**Table 4:** Comparison results among DGA module and different weight functions in terms of AUC, ACC, SEN, SPE.

Method	AUC	ACC	SEN	SPE
Max-pooling	0.693 ± 0.013	0.612 ± 0.008	0.635 ± 0.007	0.594 ± 0.018
Mean-pooling	0.712 ± 0.021	0.683 ± 0.001	0.712 ± 0.035	0.853 ± 0.002
Attention-based	0.765 ± 0.135	0.701 ± 0.100	0.761 ± 0.092	0.732 ± 0.001
DGA	**0.917 ± 0.015**	**0.904 ± 0.001**	**0.858 ± 0.008**	**0.921 ± 0.016**

All values in bold represent the optimal performance.

### 4.2 Different combination of MRI sequences

**Figure 4:**
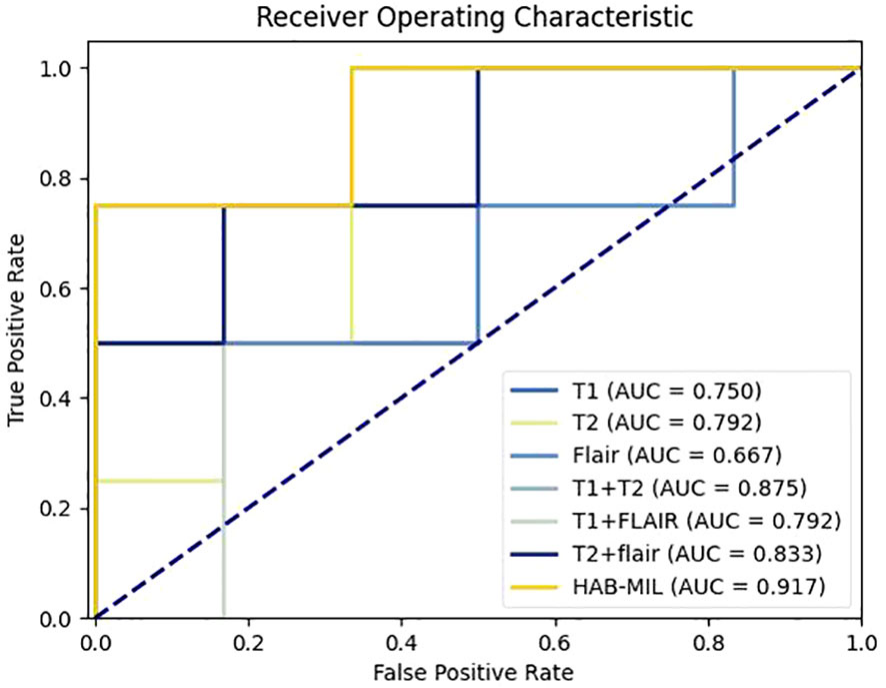
ROC curves of different sequences in the TCIA dataset.

We designed experiments to demonstrate the necessity of using different sequences simultaneously in the model. As shown in **Tables 5** and **6**, removing the T1w or T2w sequence from the input of the model results in a decrease in IDH prediction performance. **Figure 4** shows the ROC curves of different sequences in the TCIA dataset. This indicates that the T1w and T2w sequences are crucial for the IDH prediction task, as they guide the network to focus more on the tumor region and extract features that are highly relevant for gliomas.

**Table 5:** Classification results in the TCIA dataset of three different sequences in terms of AUC, ACC, SEN, SPE.

Method	AUC	ACC	SEN	SPE
T1	0.583 ± 0.135	0.502 ± 0.031	0.623 ± 0.528	0.175 ± 0.291
T2	0.667 ± 0.161	0.680 ± 0.003	0.608 ± 0.060	0.730 ± 0.012
FLAIR	0.625 ± 0.032	0.658 ± 0.031	0.637 ± 0.012	0.708 ± 0.139
T1+T2	0.854 ± 0.010	0.711 ± 0.021	0.692 ± 0.211	0.789 ± 0.091
T1+FLAIR	0.792 ± 0.164	0.820 ± 0.012	0.703 ± 0.001	0.838 ± 0.032
T2+FLAIR	0.833 ± 0.021	0.767 ± 0.121	0.675 ± 0.230	0.776 ± 0.021
HAB-MIL	**0.917 ± 0.015**	**0.904 ± 0.001**	**0.858 ± 0.008**	**0.921 ± 0.016**

All values in bold represent the optimal performance.

**Table 6:** Classification results in the in-house dataset of three different sequences in terms of AUC, ACC, SEN, SPE.

Method	AUC	ACC	SEN	SPE
T1	0.523 ± 0.031	0.561 ± 0.218	0.612 ± 0.015	0.616 ± 0.102
T2	0.576 ± 0.005	0.583 ± 0.035	0.638 ± 0.005	0.593 ± 0.001
FLAIR	0.608 ± 0.032	0.672 ± 0.010	0.665 ± 0.012	0.656 ± 0.002
T1+T2	0.761 ± 0.013	0.831 ± 0.105	0.791 ± 0.019	0.753 ± 0.052
T1+FLAIR	0.733 ± 0.031	0.793 ± 0.005	**0.812 ± 0.324**	0.792 ± 0.168
T2+FLAIR	0.833 ± 0.012	0.809 ± 0.006	0.762 ± 0.109	0.837 ± 0.020
HAB-MIL	**0.892 ± 0.031**	**0.857 ± 0.012**	0.791 ± 0.003	**0.848 ± 0.131**

All values in bold represent the optimal performance.

### 4.3 Comparison with the state-of-the-art algorithms

To validate the proposed HAB-MIL algorithm for IDH statusclassification more effectively, we compared our proposed HAB-MIL algorithm with eight state-of-the-art IDH prediction methods, including Radiomics approaches (34–36), MDL (37), MultiGeneNet (38), FAD (39), SGPNet (40), PS-Net (41), MFEF Net (24), MTTU Net (42), MTS-UNET (43) and GLISP (44). The results of the different methods are shown in the **Table 7**. By comparing the proposed method with other IDH prediction networks, the following results can be observed.

**Table 7:** Performance of all classifiers on testing set.

Model	AUC	ACC	SEN	SPE
He	0.873 ± 0.050	0.876 ± 0.090	0.875 ± 0.110	0.877 ± 0.150
Bumes	0.820	0.761	0.826	0.727
Kandalgaonkar	0.890	0.890	0.800	0.030
VGG16 ^a^	0.718 ± 0.131	0.684 ± 0.172	0.725 ± 0.231	0.606 ± 0.108
DenseNet ^b^	0.791 ± 0.324	0.838 ± 0.291	0.776 ± 0.162	0.701 ± 0.031
ResNet-50 ^c^	0.732 ± 0.002	0.714 ± 0.015	0.782 ± 0.184	0.584 ± 0.320
Inception-v3 ^d^	0.706 ± 0.272	0.668 ± 0.069	0.634 ± 0.142	0.596 ± 0.165
MDL (37)	0.872 ± 0.039	0.812 ± 0.021	0.740 ± 0.003	0.849 ± 0.001
MultiGeneNet (38)	0.886 ± 0.001	0.835 ± 0.035	0.755 ± 0.058	0.833 ± 0.036
FAD (39)	0.801 ± 0.031	0.767 ± 0.116	0.774 ± 0.236	0.734 ± 0.021
SGP Net (40)	0.837 ± 0.056	0.785 ± 0.165	0.794 ± 0.003	0.685 ± 0.355
PS-NET (41)	0.821 ± 0.013	0.791 ± 0.067	0.789 ± 0.147	0.773 ± 0.236
MFEF Net (24)	0.856 ± 0.102	0.802 ± 0.102	0.837 ± 0.312	0.813 ± 0.052
MTTU Net (42)	0.903 ± 0.056	0.857 ± 0.099	0.812 ± 0.069	0.894 ± 0.128
MTS-UNET (43)	0.803 ± 0.010	0.868 ± 0.044	0.813 ± 0.028	0.796 ± 0.030
GLISP (44)	0.750 ± 0.028	0.720 ± 0.039	0.590 ± 0.037	0.680 ± 0.106
HAB-MIL	**0.917 ± 0.015**	**0.904 ± 0.001**	**0.858 ± 0.008**	**0.921 ± 0.016**

a Visual Geometry Group 16-layer network.

b Densely Connected Convolutional Network.

c Residual Network 50-layer.

d Inception Version 3.

All values in bold represent the optimal performance.

He, Bumes, and Kandalgaonkar et al. applied radiomics-based approaches in their studies. In terms of data acquisition, Bumes utilized magnetic resonance spectroscopy (MRS), while He employed contrast-enhanced T1-weighted imaging (T1C). Although multimodal data were incorporated, insufficient feature standardization or inter-modality registration may have hindered the effective integration of information, resulting in amplified noise and reduced model performance. Moreover, conventional radiomics methods are limited to extracting shallow, handcrafted features, which may fail to capture the complex pathological heterogeneity underlying gliomas.

Firstly, GLISP model for patch-level prediction employs a lightweight CNN architecture. While this design ensures computational efficiency, it may fall short in capturing deeper and more complex pathological features compared to more advanced models. Secondly, the prediction accuracy of MultiGeneNet is higher than that of FAD. This is because MultiGeneNet is designed to simultaneously predict multiple key genetic mutations. It employs a shared feature extraction backbone with separate classification branches for each task, enabling effective feature sharing while preserving task-specific distinctions—ultimately improving overall performance. However, the model processes patch samples from whole-slide images without explicitly modeling intra-tumoral heterogeneity, which may result in the loss of important regional variations within the tumor.

Then MTS-UNET architecture lacks an explicit positional encoding mechanism, which may hinder its ability to capture spatial context—such as the relative positioning of lesions—particularly in scenarios involving high tumor heterogeneity. The poor performance of MTDL may be due to the loss of information on intra-tumoral heterogeneity and the variation in tumor size between patients. The model searches for the largest tumor bounding box and then crops the input image to a fixed large size without incorporating the tumor mask information. This approach can introduce irrelevant background information into the model, particularly when the tumor size is small.

Although the MFEF net model incorporates advanced modules such as segmentation-guided feature extraction, asymmetric amplification, and dual-attention feature fusion, the asymmetric amplification module may amplify not only pathological features, but also noise. Additionally, the feature spaces of the T2w and Flair sequences may differ significantly. Calculating these differences directly can introduce inconsistencies, affecting the stability of feature representation and prediction performance, resulting in suboptimal outcomes. MTTU Net achieved good results using a CNN transformer encoder, but it employs an uncertainty-aware pseudo-label selection method to generate and select pseudo-label. If the initial model generates a significant number of incorrect pseudo-labels, these errors may propagate and amplify with each iteration, causing the model to gradually deviate from the correct target and fall into a negative feedback loop.

Hence, the proposed HAB-MIL model is designed to address the limitations of approaches by explicitly accounting for the high heterogeneity of gliomas. It integrates positional encoding during the feature extraction phase and leverages the DGA module to emphasize the significance of key instances. As a result, it achieves superior performance in IDH status prediction compared to all other evaluated methods.

### 4.4 Interpretative visualization

**Figure 5:**
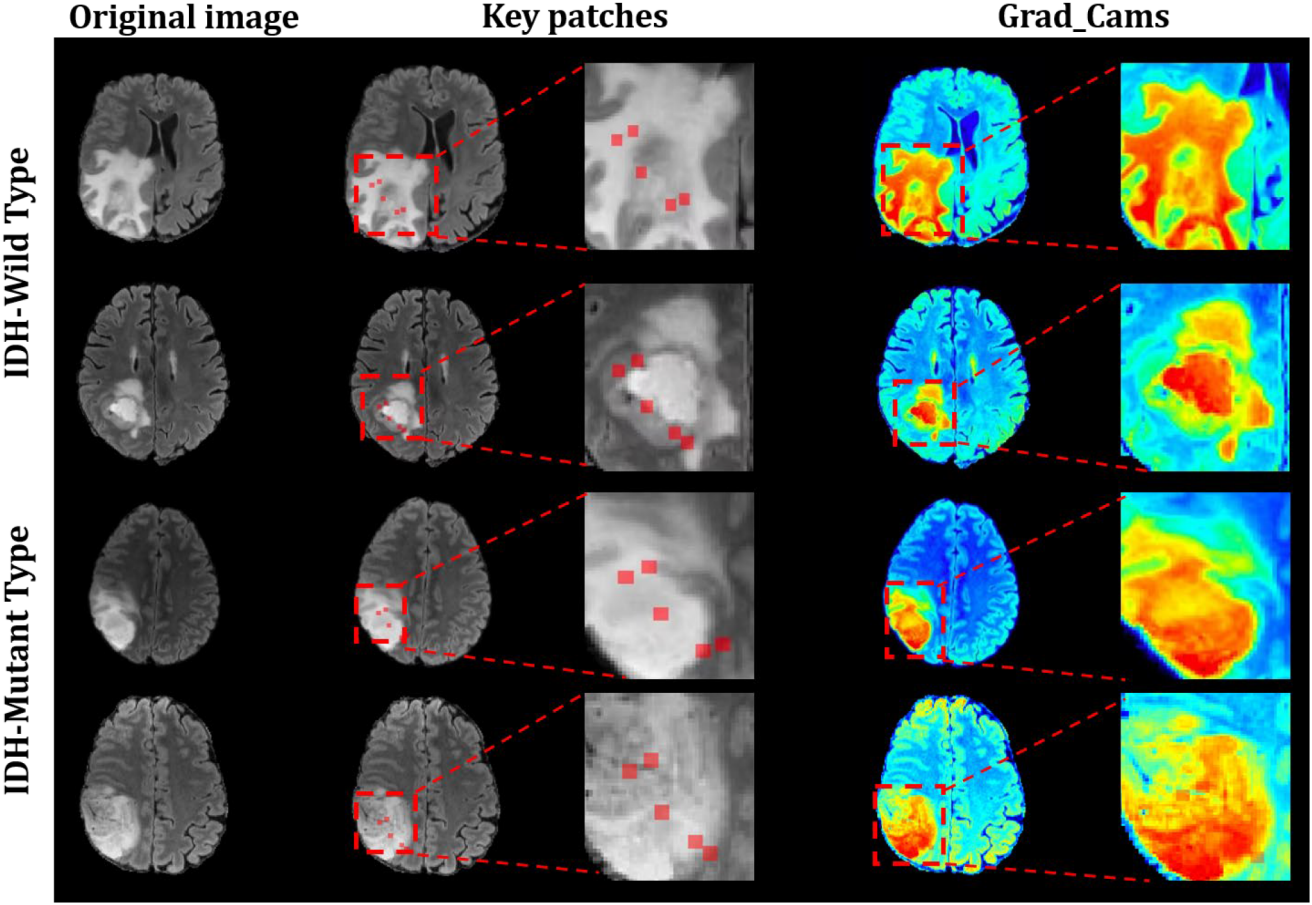
Interpretability of the HAB-MIL model. The first column is FLAIR slices extracted from different patients. Meanwhile, the second column is key patches from HAB-MIL, whereas the third column is their corresponding Grad-CAMs with the HAB-MIL.

**Figure 5** illustrates how our proposed HAB-MIL model localizes discriminative regions for the prediction of IDH status. In particular, the three columns of images are shown for each subject: (1) Original image of different patients, (2) key patches identified by HAB-MIL, and (3) Grad-CAM visualizations produced by HAB-MIL. According to **Figure 5**, the Key patches in wild-type IDH tumors are more widely distributed, encompassing both the tumor core and surrounding regions. In contrast, key patches in mutant-type IDH tumors are predominantly confined to the tumor core or adjacent areas, indicating a more localized distribution and reduced invasiveness.

The boundaries of wild-type IDH tumors appear relatively indistinct, and some tumor regions show low contrast to surrounding tissues, suggesting a tendency to invasive growth. In comparison, mutant-type IDH tumors show well-defined boundaries, concentrated lesions, and more prominent high signals in the tumor core, indicating a denser internal structure. These observations highlight significant differences in morphological characteristics, invasiveness, and growth patterns between the two types of tumors, offering valuable imaging-based insights to guide treatment decisions and prognosis assessment.

## 5 Discussion

Despite the significant success of deep neural networks in the field of IDH prediction, it still encounters challenges related to weak interpretability and low credibility. In clinical practice, classification of IDH status is crucial for guiding subsequent treatment strategies and predicting the prognosis of patients. Currently, the only way to determine IDH status is to obtain pathological tissue by open surgery. Consequently, if we can describe the imaging distinctions between IDH-wild and IDH-mutant and integrate these characteristics into the diagnostic model, the interpretability of the model would be enhanced, and its classification performance could be further improved. Following this strategy, a novel Hierarchical Attention-Based Multiple Instance Learning (HAB-MIL) framework is proposed. To effectively capture tumor location information, auxiliary positional encoding was employed to encode imaging features, which were then concatenated with the feature maps derived from a deep instance-level feature extractor, as illustrated in **Figure 1**. Tumor location, serving as a non-invasive biomarker, not only significantly enhances the accuracy of the classification model but also provides valuable insights for preoperative prediction of IDH status. Additionally, we developed a weakly supervised learning-based classification network, which substantially reduces annotation costs and better accommodates the spatial heterogeneity of gliomas. This approach facilitates the automatic identification of key regional features closely associated with IDH prediction, thereby improving the model’s accuracy, generalization performance, and clinical interpretability.

**Table 2** presents the results of the ablation study on different modules. As shown in table, the model’s performance improves to a certain extent with the addition of both modules compared to using the backbone alone. **Table 3** presents the ablation study results for different positional encoding methods. Testing with various positional encodings led to slight variations in the performance of the proposed algorithm, demonstrating the flexibility and effectiveness of GeLU. **Table 4** compares the performance of different attention mechanisms. The dynamic attention mechanism used in this study improves the precision of feature selection through a gating mechanism and dropout regularization, while also enhancing the model’s generalization capability. This approach is particularly well-suited for applications in few-shot learning or multi-instance learning scenarios.

**Tables 5** and **6** present the results of different modality combinations on the TCIA dataset and the in-house dataset, respectively. The performance on the TCIA dataset is slightly better than that on the in-house dataset, likely due to the strict quality control typically applied to public datasets prior to release. In contrast, the quality of in-house data may be affected by factors such as the acquisition environment, equipment variability, or human error. In both datasets, the combination of three modalities consistently yields better performance, which attributed to the varying sensitivities of different modalities to tissue structures, lesions, and fluids. By integrating multiple modalities, more comprehensive and informative imaging data can be obtained. Additionally, the network shows increased robustness to missing modalities when setting the T1 and T2 or FLAIR sequence to zero while training. There is only a small decrease in performance when only providing the T1w, T2w and Flair scans compared to all three MRI as input. This is especially useful as not all three MRI modalities are available for all patients in the Xi’an Jiaotong University Hospital dataset. This way an accurate prediction could still be obtained for these patients. The very high specificity and slightly lower sensitivity indicate that prediction inaccuracies are due to parts of the surrounding edema that are not detected by the network.

An in-depth analysis of **Figure 4** and the attention weights learned by the model reveals a consistent anatomical distribution pattern of lDH-mutant gliomas. Specifically, gliomas located in the thalamus and cerebellum are predominantly IDH wild-type, whereas those in the insular cortex are more likely to harbor IDH mutations. This observation is consistent with previous radiological and histopathological studies, further validating the reliability of our model. Compared to existing methods, our approach offers three distinct advantages: first, HAB-MIL accurately identifies subtle imaging differences between IDH wild-type and lDH-mutant gliomas through key instance selection, helping clinicians pinpoint regions that are most informative for distinguishing IDH status. Second, the process of identifying key instances is intuitive and straightforward to implement. Finally, the clinical application of this model requires only routine MRI sequences (T1w, T2w, and FLAIR) as input, without the need for contrast agents, thereby reducing potential risks in practical use. Furthermore, the model processes the entire MRI image directly, requiring only skull-stripping and registration steps, without manual tumor delineation by radiologists. This design minimizes reliance on specialized expertise and facilitates broader clinical implementation. This study still has several limitations. First, the HAB-MIL model has not yet been validated in large-scale, multicenter clinical settings; therefore, its feasibility and stability in real-world clinical practice remain to be further confirmed. Second, some cases in this study lacked key molecular markers such as TERT promoter mutation, EGFR amplification, and chromosome 7 gain/chromosome 10 loss (+7/−10).

## 6 Conclusion

In this study, we proposed a HAB-MIL framework, contributing to the prediction of IDH status using only routinely acquired preoperative MRI. Compared with conventional clinical workflows that require labor-intensive, slice-by-slice tumor annotation, HAB-MIL employs a weakly supervised learning strategy that relies solely on case-level labels, eliminating the need for detailed lesion delineation. This approach significantly reduces annotation time and manual effort while preserving model accuracy. Moreover, the model requires only routine MRI sequences such as T1-weighted, T2-weighted, and FLAIR images, without the need for contrast agents, thereby minimizing potential procedural risks and reducing the overall financial burden on patients.

In addition, future work will focus on further refining and expanding this study. First, we plan to validate the HAB-MIL model in large-scale, multicenter clinical settings to evaluate its feasibility and stability in real-world clinical practice. Second, we will collect more diverse datasets encompassing cases acquired from different imaging devices and scanning protocols, as well as additional modalities such as MR perfusion and DTI, to further enhance the model’s generalization and robustness. Finally, we will conduct comparative analyses with other noninvasive prediction methods to comprehensively evaluate the model’s performance.

## Data Availability

The original contributions presented in the study are included in the article/supplementary material . Further inquiries can be directed to the corresponding author.
